# Unplanned admissions and the organisational management of heart failure: a multicentre ethnographic, qualitative study

**DOI:** 10.1136/bmjopen-2014-007522

**Published:** 2015-10-19

**Authors:** Rosemary Simmonds, Margaret Glogowska, Sarah McLachlan, Helen Cramer, Tom Sanders, Rachel Johnson, Umesh Kadam, Daniel Lasserson, Sarah Purdy

**Affiliations:** 1 Centre for Academic Primary Care, NIHR School for Primary Care Research, School of Social and Community Medicine, University of Bristol, Bristol, UK; 2 Nuffield Department of Primary Care Health Sciences, University of Oxford, Radcliffe Observatory Quarter, Oxford, UK; 3 Arthritis Research UK Primary Care Centre, Research Institute for Primary Care & Health Sciences, Keele University, Keele, UK; 4 Section of Public Health, ScHARR, University of Sheffield, Keele, UK; 5 NIHR Oxford Biomedical Research Centre, John Radcliffe Hospital, Headley Way, Oxford, UK

**Keywords:** PRIMARY CARE

## Abstract

**Objectives:**

Heart failure is a common cause of unplanned hospital admissions but there is little evidence on why, despite evidence-based interventions, admissions occur. This study aimed to identify critical points on patient pathways where risk of admission is increased and identify barriers to the implementation of evidence-based interventions.

**Design:**

Multicentre, longitudinal, patient-led ethnography.

**Setting:**

National Health Service settings across primary, community and secondary care in three geographical locations in England, UK.

**Participants:**

31 patients with severe or difficult to manage heart failure followed for up to 11 months; 9 carers; 55 healthcare professionals.

**Results:**

Fragmentation of healthcare, inequitable provision of services and poor continuity of care presented barriers to interventions for heart failure. Critical points where a reduction in the risk of current or future admission occurred throughout the pathway. At the beginning some patients did not receive a formal clinical diagnosis, in addition patients lacked information about heart failure, self-care and knowing when to seek help. Some clinicians lacked knowledge about diagnosis and management. Misdiagnoses of symptoms and discontinuity of care resulted in unplanned admissions. Approaching end of life, patients were admitted to hospital when other options including palliative care could have been appropriate.

**Conclusions:**

Findings illustrate the complexity involved in caring for people with heart failure. Fragmented healthcare and discontinuity of care added complexity and increased the likelihood of suboptimal management and unplanned admissions. Diagnosis and disclosure is a vital first step for the patient in a journey of acceptance and learning to self-care/monitor. The need for clinician education about heart failure and specialist services was acknowledged. Patient education should be seen as an ongoing ‘conversation’ with trusted clinicians and end-of-life planning should be broached within this context.

**Strengths and limitations of this study**

- This qualitative study on the management of heart failure (HF) is unique in providing detailed accounts of patient pathways across service providers from multiple perspectives and in highlighting structural and individual factors that can create barriers to services or increased risk of hospital admission.
- The findings from this study are of relevance to a range of clinicians as well as commissioners and service managers. The provision of person-centred care for people with heart failure is an important message that underpins our findings.
- A methodological strength of this study was the ethnographic approach, which enabled documentation of healthcare in real time, rather than through recall. Triangulation of data by location, ethnographers and qualitative methods increased the trustworthiness of findings. Issues arising from awareness of methodological and researcher reflexivity were addressed by the research team throughout the study.
- Limitations of this study included the framing of unplanned hospital admissions as problematic—a perception that was not shared by some participants. Being observed by a researcher did alter clinician behaviour in some instances but descriptions of ‘usual’ care were provided by patients as a comparison.
- A limitation of this study was the small sample size of 31 patients, which may not represent the diversity of people with severe HF. However, we feel the transferability of our findings can be assessed by their credibility, and concordance with existing literature.

## Introduction

Unplanned admissions to hospital are expensive for healthcare systems, and are distressing for patients and their families. Heart failure is a complex condition and patients are frequently admitted to hospital due to exacerbations of symptoms.^1–3^ In England there were 68 654 such admissions in 2012/2013^4^ with a total overall cost of around £119 million per annum. The average cost of a non-elective inpatient admission for heart failure was £2231 in 2010^5^ with 12.2 days being the mean length of stay. Risk of readmissions for patients with heart failure is high at around 16%.^4^

Multidisciplinary approaches, delivered in the community and specialist clinics^6^ have been shown to be effective in improving the management of heart failure and in reducing the risk of emergency admissions. The use of multidisciplinary, community-based approaches are also reflected in current clinical guidelines.^7^ Other interventions that are effective in reducing admissions for patients with heart failure include appropriate utilisation and dosage of medication,^7^ medication reviews,^8^ case management on discharge from hospital,^9^ patient education^10–12^ and end-of-life care.^13^

In studies involving long-term conditions, continuity of care has also been raised as an important influence on admissions.^11^ ^14–16^ One framework of continuity makes a distinction between continuity of relationship (a continuous caring relationship with clinicians) and continuity of management (all aspects of integration, coordination and sharing of information).^16^ Relational continuity is associated with lower rates of emergency department attendance, and hospital admissions^17^ ^18^ and has been associated with improved outcomes for patients, particularly in preventive care and medication adherence.^19–22^

Previous qualitative studies have provided some insight into factors which may underlie unplanned hospital admissions in patients with heart failure. For example, a qualitative meta-synthesis of literature on help-seeking in heart failure patients identified a number of barriers to timely access to treatment, including uncertainty over the need for help-seeking with fluctuating symptoms, uncertainty about who to contact, fear of hospitals and patients’ attribution of symptoms to other causes.^23^ However, studies have mainly focused on patient factors, such as self-care,^24^ knowledge^25^ and adherence to treatment^26^ or healthcare professional factors, for instance beliefs and attitudes^27^ ^28^ and experience in managing patients with heart failure,^29^ in isolation, with a dearth of qualitative literature exploring how these different factors may interact. The impact of caring for a patient with heart failure places considerable burden on informal carers, particularly in those patients with advanced heart failure who are coming to the end of life.^30^ ^31^

Although there is evidence on factors associated with heart failure admission and interventions that link types of care provision with the reduction of admissions in heart failure, to our knowledge there are no existing studies exploring why admissions continue to be common. Previous qualitative studies in heart failure have largely relied on interviews and focus groups. To develop an understanding of the interactions between patient, carer, healthcare professional and system factors in unplanned hospital admissions, an ethnographic approach was adopted. Ethnography is defined as “the study of social interactions, behaviours, and perceptions that occur within groups, teams, organisations and communities”^32^ (p.512). Ethnographic research usually involves observing people in *their* real-world settings and is therefore particularly useful for determining what people do in a context, rather than what they might say they do in more contrived research settings, such as in focus groups or interviews. An ethnographic approach can provide valuable insights into what people need in their everyday or professional lives when experiencing illness or delivering healthcare. For these reasons we decided an ethnographic approach was most suited to achieving the aims of the study—that is to identify critical points on pathways where risk of admission is increased and barriers to the implementation of evidence-based interventions.

## Methods

### Participants and recruitment

The inclusion criteria were adult patients with an unplanned hospital admission for heart failure during the preceding 6 months and who the referring clinician considered had severe or difficult to manage heart failure (with or without physical or mental health comorbidities). Our sample size was determined by the aims of the study which necessitated an in-depth methodology. Ethnographic fieldwork can be time consuming and provide significant amounts of data, so our sample size also reflected the practicability of the research design together with the desire to maintain qualitative rigour.

Patients were recruited via general practitioner (GP) practices (sampled for a range of practice level social deprivation scores and rurality) specialist nurses and secondary care-based services, including two teaching hospitals, across three study sites. The three study sites were a mix of urban and rural settings covering large geographical areas and with variable access to heart failure specialist nurse-led clinics Potentially eligible participants were identified at one site by screening of patients on the hospital ward or in heart failure clinics and at the other two sites by healthcare professionals in heart failure clinics and general practices. Patients at the first site were then approached directly in person by a health services researcher external to the study who invited patients to consider participation in the study. At the other two sites potential participants identified from heart failure clinics and general practices were sent letters of invitation. If potential participants at all three sites indicated they were interested in the study they were then contacted by the study team. Overall, of the patients who gave their consent to being contacted by the study team, 13 declined to participate. Three patient participants died during the course of the study. Informal carers of recruited patients were invited to participate. Participation in the study ranged from 1 to 11 months. Written informed consent was obtained from all participants by the researchers who were not known to participants.

### Data collection

Three social scientists (RS, SM, MG) carried out all data collection. Participating patients were followed individually using ethnographic methods (observation, impromptu interviews) throughout their interactions with healthcare, for a period of up to 11 months during 2011–2013

In-depth interviews were planned with a subsample of patients or carers/family members (around eight at each site), to include around four patients or their carers experiencing exacerbations and hospital admissions for heart failure and four without hospital admissions during the follow-up period (thereby including different patient trajectories and severities). Recorded fieldwork conversations (impromptu interviews) with patients, carers and health professionals were conducted and analysed as an integral part of the ethnographic fieldwork.

Interviews with clinicians covered topics of: their role and experience of patients with heart failure; their perceptions of what constituted an avoidable admission; why patients are admitted to hospital; challenges for clinicians and people with heart failure in managing the condition and recommendations for improvements that might reduce admissions. The majority of healthcare professionals in the study were caring for study participants and were observed delivering care. A minority of health professional participants were caring for people with heart failure who were not participating in the study. These healthcare professionals took part in prearranged interviews about their general experiences of caring for people with heart failure and what might trigger an unplanned hospital admission.

Interviews with patients/carers explored their accounts of living with heart failure and what they perceived as key events in the illness and care received. Impromptu fieldwork interviews examined participant perspectives on events as they were happening—questions focused on the specifics of the event. The interviews took place in primary and secondary healthcare settings and patient’s homes.

Although topic guides were used for patient and clinician in-depth interviews, participants were able to speak freely about their experiences and raise topics not covered by the guides. Topic guides were developed based on a review of the relevant literature, expert advice from the independent Study Advisory Group and key informant interviews with staff involved with the management of patients with heart failure. Interviews were audio recorded and transcribed verbatim.

In observing healthcare, researchers accompanied most patients to healthcare appointments where perceptions, priorities and interactions with clinicians were explored/observed. Observational data were recorded in notebooks and audio recordings. Documentary data comprised patient and carer diaries and included information from patient primary care and secondary care medical records comprising consultations, investigations, medication and correspondence relating to admissions and outpatient appointments, which were collected for analysis at the end of study participation. Four patients and 6 carers agreed to write diaries. Diaries were completed for a period of between 3 weeks and 10 months. Participants could choose to opt out of this task at any point. There were two topic guides posing questions for the diarist to address, one for carers and one for patients. For patients we asked for example: what was the health or social care received that day; what was the purpose of the treatment/care; what worries them the most.

### Analysis

All data were analysed using an inductive, thematic approach^33^ involving a process of constant comparison between cases^34^ and were supported by the use of NVIVO V.10 qualitative software programme to aid management and analysis of data. Data were stored on a secure server. Analysis began alongside data collection. Researchers added analytical and reflexive comments to field notes immediately after observations. Ideas from early analysis informed later data collection in an iterative process. Analysis of individual transcripts, observational data and documentary materials started with open coding grounded in the data. This generated an initial coding framework, which was added to and refined, with material regrouped and recoded as new data were gathered. Codes were gradually built into broader categories through comparison across transcripts/field notes/documents and higher-level recurring themes were developed.

Observational data, impromptu/fieldwork interviews and documentary materials were analysed at three levels (1) individual patient cases (2) across cases within research centres (3) across research centres to synthesis. Thematic analysis of these data was aided by ‘Situational Analysis’—a grounded theory approach involving mapping of patient/carer experiences and the organisation of healthcare systems.^35^

Credibility and trustworthiness^36^ of the data (qualitative rigour) was achieved by ‘member checking’ initial analytical ideas with both our research participants (iteratively) and our patient/carer advisory group. Coding frameworks, disconfirming views and the development of final themes were discussed regularly by the multidisciplinary research team. Data triangulation via different researchers, locations and qualitative methods was used to corroborate analytical themes. The external validity of our findings was enhanced by reference to existing social theory and relevant conceptual frameworks.

The first stage of data triangulation was at an individual patient level. Data from professionals, carers and patients were compared systematically across all transcripts and common narratives were used to inform the thematic analysis. The data were used together as part of an integrated thematic analysis where interview quotations and observational data were grouped under the relevant themes and compared for similarities and differences. This way we were able to identify common themes from patients and healthcare professionals, highlighting similarities and differences in relation to the care of patients with heart failure. Conflict of opinion/views was treated as a naturally occurring finding highlighting the diversity of perspectives and the complexity of chronic illness management. The study did not seek to capture ‘factual’ information as such, and aimed to examine the richness of views and the complexity of clinical and patient-related decision-making.

We used chronological charts to track and compare events and issues in patient and carer stories arising from field notes, interviews and diaries, with patients’ medical record event ‘stories’. Events in patients’ medical records and related documents were summarised and entered into a chronological chart which was divided into two columns, one for the patient/carer story and one for the medical story. We examined and contrasted individual patient/carer accounts with their medical record accounts, and identified and discussed any dissonance between these. We then combined the key elements of personal and medical stories into situational maps for each patient (following methods outlined by Clarke in 2005)^35^ and examined the maps in relation to five questions: who and what are in the situation; who and what matters in the situation; what elements make a difference in the situation; what are the physical triggers for admission; what can tip the balance for admission. After synthesising key themes from the situational maps, within each research centre, the second stage of data triangulation involved comparison across the three research centres with input from our patient/carer advisory group and professional advisory group.

Structuration theory was used as a sensitising device to inform the analysis.^37^ Structuration theory suggests that to understand the social world we need to look at the role of both individual actions and structural or organisational factors which shape how individuals behave, and which influence each other iteratively. In our study this meant we needed to examine both how individual clinicians and managers behave, but also how their actions are determined or constrained by the way local services are organised and funded (eg, broader policy and organisational procedures/directives). To operationalise the basic tenets of structuration theory, we used the concept of continuity of healthcare at managerial (structural/organisational) and relational (individual) levels.^16^

## Results

We recruited 31 patients, 9 informal carers and 55 healthcare professionals. Sixteen patients were male, 10 patients lived alone, 5 lived in deprived areas and the average age was 72 years. The majority of patients described their ethnicity as White British. Twenty three clinicians participated in in-depth interviews across research sites including: 7 GPs; 4 community nurses; 5 heart failure specialist nurses; 5 senior hospital doctors (including 3 consultant cardiologists) and 2 cardiac rehabilitation therapists.

The final data sets comprised over 100 h of observation, 44 recorded impromptu interviews with patients, carers and clinicians, 10 patient and carer diaries (four patients and 6 carers*)*, 18 patient medical records, 22 patient and/or carer in-depth interviews and interviews with 24 clinicians. All patients (and carers where they participated) were interviewed about the patient's ‘heart failure journey’ at the beginning of participation in the study. Some of these interviews were in-depth interviews and others were shorter ‘impromptu’ interviews. All patients were also invited to take part in an exit interview. No patients declined to participate in an exit interview but not all were available or able to do this. Additional impromptu interviews were then conducted at different points in the study. All admissions were recorded but only those directly related to heart failure were included in this analysis.

From these data we identified barriers to accessing services/interventions aimed at reducing admissions for heart failure and critical points in the patient pathway for unplanned admissions. Issues were identified in the organisation of healthcare systems and in the delivery of clinical care.

### The organisation of healthcare systems: identifying barriers to service utilisation

#### Organisational fragmentation

This theme relates to how fragmented and incoherent services impacted on the accessibility, quality and continuity of care for patients with heart failure. The issue of service fragmentation was present in varying degrees across the three study centres with Centre A having the most fragmented healthcare system.

**Figure 1:**
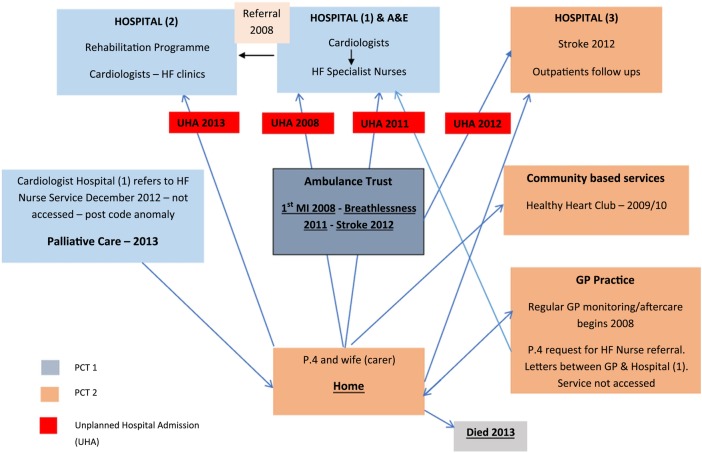
Diagram illustrating healthcare journey for P.4, Centre A, from 2008 to 2013.

To illustrate how service fragmentation impacted on care at Centre A, we refer to the experiences of participant 4 and carer, as an exemplar. This patient's home location and GP practice straddled the boundaries of two different primary care trusts (PCTs) and three hospital trusts. A diagram of this participant's healthcare journey (figure 1) shows care provided by multiple providers. There were four unplanned hospital admissions at three different hospital trusts and a failed referral to the heart failure specialist nurse service. Poor managerial and relational continuity of care resulted in lost referrals, cancelled appointments and delayed access to specialist healthcare (box 1A). These experiences were shared by several patients at Centre A.

Box 1 **Organisation of healthcare: Identifying barriers to service utilisation**

Organisational fragmentation

- P.4 had a heart attack in 2008…he was taken to the A&E at Hospital (1). A stent was inserted….Hospital (1) told him that they would not be able to see him after discharge and that he would be followed up with Cardiology outpatients appointments at Hospital (2). Five months passed and P.4 didn't hear anything from the 2nd hospital—apparently his paperwork had not been passed on. When he did attend, Cardiologist was not aware of him so he had to go through a post-op check-up procedure. He started to have 6 monthly appointments but then the hospital kept cancelling them and on one occasion accused P.4 of cancelling an appointment (which he didn't do). It has been 12 months since he has seen a Cardiologist. (Patient 4, observation field notes, Centre A)
- Researcher: …how important do you think in your overall picture that the absence of the notes has been…?
  -  *Participant*: Well I'm sure I would have had a pacemaker fitted earlier.
  -  *Outpatients appointment with cardiologist (Hospital 2)*
  -  …the last appointment he had for his “guts” was at [name of community hospital] but his notes did not arrive. Clinic Nurse apologises and says his notes have not arrived at Hospital 2 and they have been trying to locate them since 9 a.m. this morning—they are on their way from [name of Community Hospital] (these arrived after his appointment). Mrs P.5 [Carer] says that his notes did not arrive at his last Cardiology outpatients’ appointment in February, either.
  -  Went to gastro man about bloating—NO NOTES. Mrs S has put in formal complaint to [Hospital 2] about missing notes via NHS complaints procedure. They have acknowledged complaint and promise to give written response by 30.11.12. (P5, telephone call field notes, Centre A)
  -  Mrs P.5 told Cardiologist [Hospital 2] that she had written a letter of complaint to the Trust about her husband's notes not turning up for his appointments and posing the question of how this might affect his healthcare if there weren't notes to refer to?…Mrs P.5 tells me about a [subsequent] surprise urgent appointment with Cardiologist at Hospital 2 on 29/11/12. P.5 was told that the results of the 48 h monitor (February/March 2012) showed his heart was going slower than it should at 30 something and he should be referred for an ICD …Mrs P.5 was speculating on there being a connection between the complaint to the Trust and the urgent appointment. Mr & Mrs P.5 wonder if the results of this test had not been looked at or had fallen to the bottom of a pile and then the official complaint brought them to light.
  -  *Cardiology outpatients’ appointment at Hospital 2*—arrived at outpatients’ clinic and met Mr & Mrs P.5…There were NO NOTES!!
  -  (Patient 5, extracts from fieldwork interview and observation field notes, Centre A)

Access to specialist services

- …well they gave me numbers and information but every time I tried to ring [acute heart failure clinic] …(left messages) never actually speaking to anybody, in the end nothing ever came of it… Yeah he [hospital heart failure nurse] said I think you’d benefit from coming to the clinic…(Patient 3, fieldwork interview, Centre B)
- At the moment we're picking up about 70% of all the heart failure admissions, but a lot of them are discharged within 48 h so if they do come in on Friday afternoon and we don't know about them until Monday morning we might have missed them. (HP12, interview, heart failure specialist nurse, Centre C)
- …because the medical teams are so busy with the acute medical take, they just do not have time always, nor do they think of referring patients to the heart failure team and so we have to find them ourselves which involves trawling through the admission lists… (HP11, interview, cardiologist, Centre C)
- …we were asked if we wanted to see the heart failure nurse and we said ‘yes.’ The problem was that the GP tends to look after one side of the heart and the heart failure nurse looks after the other…and it was the wrong side of the heart…so…we haven't seen the heart failure nurse. (Carer to P6, interview, Centre C)
- Well I think the heart failure specialist nurse service is excellent, because they're very caring people, when they take on a patient they go the extra mile. And I think the problem is they can't take on enough patients, and that's partly because of the service provision being inadequate…(HP3, GP interview, Centre C)
- ([Cardiologist Hospital 1, Centre A] asks if he [patient] has seen a heart failure nurse—“no”—“you should do, you have been left out!” Cardiologist explains that there is an inequity in the service to do with where people live –“I will sort it out!” (P4, observation field notes, cardiology outpatients appointment, Centre A)

Poor management continuity, especially across the primary/secondary care interface impacted on the care of another participant in this locality. Participant 5 had multiple health conditions and was referred to a variety of different health providers. Some had incompatible IT systems, relying on paper medical records for clinical decision-making which were repeatedly lost. This patient and carer were worried about the absence of medical notes at secondary care consultations and particularly concerned that significant results from a 48 h heart monitor test had not been acted on for nearly 9 months (box 1B). The resulting delay in referral for a pacemaker could have resulted in an unplanned admission or other significant event.

The lack of continuity and barriers to obtaining care placed a particular burden on carers. Carers’ diaries and ethnographic observations highlighted the emotional impact on the carer of the patient's condition and their critical role in encouraging the patients to access health services and acting as the patient's ‘champion’ (box 1).

Our data illustrates how fragmented services and poor managerial and relational continuity of care can be seen as barriers to receiving timely clinical interventions for heart failure that can have serious consequences for patients. Although there were exemplars of well-coordinated care in some of the study research sites, our interest was in identifying barriers to the delivery of services that may reduce admissions for heart failure.

#### Access to specialist services and support for heart failure

A number of organisational barriers were identified in accessing specialist heart failure services, across the three research centres. A patient had difficulty in accessing a heart failure clinic by telephone, there was Monday to Friday provision of care and working pressures/professional norms in an acute hospital care necessitated a cardiologist having to ‘trawl’ through admissions lists to find patients (box 1C–E).

Organisational and financial factors created barriers at one centre where access to community-based, heart failure specialist nurses was restricted to patients with left ventricular systolic dysfunction (LVSD) only. In this context an account, from a carer's perspective, provides an example of physiological fragmentation and professional demarcation according to differing categories of heart failure. For this participant, the heart appears to have been divided up and given meaning as ‘right’ or ‘wrong sided’ (box 1F). In another centre, financial/organisational barriers created problems for patients in accessing heart failure specialist nurses if they lived in PCT geographical areas that did not fund the service (box 1G, H).

In addition to these barriers there was also a lack of managerial continuity in the provision of care causing confusion and frustration for patients, carers and staff. Some clinicians attempted to override these barriers and intervene in normative processes so they could refer patients to specialist services (box 1H).

### Delivery of clinical care: points in the patient pathway where risk of admission is increased

#### Disclosure of diagnosis and educating patients about heart failure

A diagnosis of heart failure, via an echocardiogram, should trigger an explanation of heart failure to the patient. This first conversation can be important for beginning a patient's personal journey of acceptance of heart failure “…for patients, this process of coming to see illness as part of the self is likely to influence how they engage with self-care behaviours” (ref. 38, p.101).

However, clinicians can find this first conversation difficult, regarding ‘heart failure’ as a loaded term, on a par with cancer, which may come as a shock to newly-diagnosed patients (box 2 A, B). To avoid upsetting patients and extinguishing hope, some clinicians talked about heart failure in euphemistic terms, such as having an ‘ageing heart’, ‘stiff heart’ or ‘heart not pumping efficiently’.

Box 2 **Disclosure of diagnosis and educating patients about heart failure**

- Well it [the term heart failure] has a negative concept to many people, because I think for a lot of patients it's an organ that's failing…therefore a sort of terminal illness… there's a lot of anxiety about that term. (*HP4, interview GP, Centre A*)
- …I think if you're given a diagnosis of cancer people automatically jump to whatever conclusions but with heart failure, even though the title is horrible and people do sometimes not like it being called heart failure, which yes, is not very nice but it is just something that everyone understands what that is in the medical world…I think if they've got other things going on they [patients] often think of heart failure as a secondary diagnosis and they're more concerned about the diabetes or other thing they've got going on, so you don't want to flatten them by telling them anything grim, but really you can't paint false hope either…” (*HP6, interview specialist nurse, Centre C*)
- So some people will be admitted to hospital for the first time, that's when a diagnosis will be made. That's not a good way to make a diagnosis…”
  - (*HP9, interview consultant elderly care, Centre A*)
- *Interviewer*:…when you went into the [name of hospital] and you first knew that you, you'd got heart failure…how was that kind of explained to you? Do you remember?
  - *Participant*: It wasn't explained a lot to me. I had to get some leaflets downstairs to read about it…” (*Patient 3, field work interview, Centre A*)
- …her husband's discharge letter had been “horrendous” …the doctor had been very direct…[and] had focused on the word “death” and [carer] found this very distressing. (Patient 4, observation field notes, Centre B)
- The patient informed me that while in hospital, he had received information only through his discharge papers and there had been no mention of the diagnosis to the patient in person. (*Patient 7, observation field notes, Centre B*)
- *Interviewer*: Hmm. [Pause] So when you were in hospital for the five days…and you said you were prescribed medication and things like that, did anybody there explain what was happening and what had caused it…did you receive any more information while you were in hospital…
  - *Participant*: Only the information that was on my discharge papers.” (*Patient 7, fieldwork interview, Centre B*)
- …we weren't given a lot of information during visits to the hospital…disappointing really…it would have been nice of them to find a member of the family and explain that, otherwise we're just left in the dark a bit… (*Carer to Patient 6, fieldwork interview, Centre C*)
- I had a chat with the heart failure nurses before I came out. They gave me a list of instructions of what to do, check your weight and things like that, which I'm doing. (*Patient 9, fieldwork interview, Centre C*)
- The nurse explained to P4 that he has a chronic condition and that there is no cure, and that the main aim is to help the patient manage. The patient's wife…said that she had felt better after speaking to the HF nurse…(*Patient 4, observation field notes, Centre B*)

Disclosure and explanation of diagnosis during the course of an unplanned hospital admission was perceived as unhelpful and inadequate by clinicians, patients and carers. Clinicians observed how a busy hospital was not conducive to the provision of appropriate explanations of heart failure (box 2C) while patients and carers reported being given very little or insensitive information about their condition during an in-patient stay (box 2D–H). However, where patients had good access to hospital and community-based heart failure specialist nursing teams, they reported more positive experiences (box 2I, J).

A lack of patient information and education was a strong theme in our study and a key barrier to the development of patient self-help strategies that can help prevent readmissions. Healthcare participants emphasised the need for information and guidance to be given to patients as part of an ongoing conversation. HF specialist nurses and GPs were seen as key to the success of this process.

#### ‘Decision flashpoints’ and the management of exacerbations in primary care

Medical record review revealed that most patients in the study also had multiple comorbidities which made managing heart failure more challenging. For a number of participants, what preceded treatment for an exacerbation of established heart failure was a period of time when they were treated for respiratory illnesses such as asthma or a chest infection. An emergency admission to hospital can be the result of ‘decision flashpoints’ where a clinical misdiagnosis of symptoms (typically breathlessness) can send the patient along a different disease pathway until they are in need of emergency in-patient care (box 3A–D). These barriers to diagnosis and appropriate care are perhaps more likely to occur when there is a lack of relational continuity with a GP (box 3E).

Box 3 **Decision ‘flashpoints’ and the management of exacerbations in primary care**

- *Participant*: You're asking me to give examples…a patient that's come through the door that's gone to their GP maybe two or three times with a chest infection, and had been given antibiotics and treated for what was a diagnosis of chest infection, and it turned out…it was heart failure, that's very common…very, very common, so had that GP or GPs …actually really looked at the patient and carried out their risk factors and carried out a chest X-ray, they may have then been able to do a diagnostic referral, and that patient may have been caught earlier on…
  - *Researcher*: Right, so could this be the reason why a patient might end up as an emergency admission?
  - *Participant*: It definitely is, definitely, definitely.” (*HP20, interview heart failure specialist nurse, Centre A*)
- He [Patient] had an unplanned hospital admission because he developed a bad cough. He was put on antibiotics and then a different course of antibiotics and then a GP (not his regular GP) took him off his heart medication for a period of three to four days. The cough got so bad that he could not breathe and he was taken into hospital. (*Patient 4, observation field notes, Centre A*)
- P.5 explained that he had felt ill when he returned from a trip to Canada…He described his symptoms as breathlessness, nausea, lethargy, dizziness and a lack of appetite. The patient saw his GP, who prescribed antibiotics. The patient returned for another appointment a fortnight later, as his symptoms had got progressively worse. The patient had oedema, could not climb the stairs at home and was producing phlegm. At the second appointment, the patient was given different antibiotics. Shortly afterwards, the patient's daughter—who is a senior paramedic—came to see her father and did a 12-lead ECG. The patient told me that his daughter had been “terrified” by the results. The patient also spoke to his other daughter, a hospital consultant, who instructed her father to go into hospital, urging him ‘you go today or you won't be here tomorrow.’ The patient's daughter felt that the GP should have done an ECG on her father when he presented at the surgery, particularly as the patient has a pacemaker. (*Patient 5, observation field notes, Centre B*)
- The patient explained that he had been diagnosed four weeks ago. The patient had not been feeling well and had experienced swollen legs. The patient had dismissed the symptoms and had carried on as usual… symptoms, including some breathlessness, had been present for about a month…At the time, the patient and his wife thought that the breathlessness related to the patient's asthma. The patient went to the doctor, who diagnosed a chest infection and prescribed an inhaler (Ventolin). When this did not relieve the symptoms, the patient saw a different GP who sent the patient for blood tests. When the patient returned to discuss the results of the blood tests the following week, the doctor said that she would call for an ambulance to take the patient to hospital. (*Patient 4, observation field notes, Centre B*)
- …one of the problems of primary care…patients they are telling me…they say they are not seeing the same GP…Sometimes they see their normal GP, sometimes they see another GP…Sometimes they see a locum GP…So this variation itself, to be honest with you it, er, it makes patients…they lose confidence…So continuity of the care actually…is important…(*Cardiologist to P.4, fieldwork interview, Centre A*)
- …if the GP's not interested in hearts, because not all GPs have got an interest in cardiac because they are generalists…so that would be one reason. Or the GP wants to treat the patient himself, he doesn't want anybody else to look after his patient or her patient, in which case you get mismanaged patients that then are re-admitting to hospital because they've never been managed and they've always been, gone back to the GP who they love, who's very good at talking but not good at underpinning care. (HP20, interview heart failure specialist nurse, Centre A)
- …my experience of heart failure nurses is fairly limited so I'm not in a position to know a huge amount about what they do…he [patient] will weigh himself every day so you know he can do a lot of that basic stuff that I think GPs are probably a little bit rubbish at doing. (*HP4, interview GP, Centre A*)

The way in which individual GPs managed patients was also perceived by some heart failure specialists to be associated with an increased risk of admission. Some GPs lacked knowledge of heart failure and specialist services (box 3F, G), resulting in ‘mismanaged patients.’ GPs could also be possessive of their patients, presenting a barrier to referral for services such as specialist nurses (box 3F).

#### End-of-life care

National Institute for Health and Care Excellence (NICE) guidelines for heart failure at end of life, recommend that patients and carers have the opportunity “at all stages of care to discuss issues of sudden death and living with uncertainty.”^39^ Not knowing when or how to broach this difficult topic with patients can result in the issue being sidestepped by clinicians (box 4A). As a result, patients are likely to be admitted to hospital unexpectedly at end of life (box 4B).

Box 4 **End-of-life care**

- That's the difficulty—knowing when to…having difficult conversations with people, because we don't like talking about end of life in this country, it's still a taboo subject, very much so. (*HP21, community matron Centre A*)
- …too many people come into hospital and die and you know that there is not that advanced care planning for people to die at home…if they've got heart failure they're going to continually get worse…and if there was enough support in the community they could stay at home…(*HP4, specialist nurse Centre C*)
- …nobody can know that with heart failure unfortunately it's really hard to know, no matter how much experience you have, you're constantly surprised by sudden death, equally…you can say this lady is at the end of her life, and then you find that 2 years later you're seeing her in clinic and you see in your previous clinic letter it says end of life, and you think oh gosh, still here…(*HP4,interview heart failure specialist nurse Centre C*)
- Hospice care for a complex patient and admission avoidance
  - I [researcher] rang to see how things were going. He [carer] said he visited his father last night and he was surprised to see him sitting up in bed, chatting to the nurses and drinking tea/eating cake. The doctor had seen him and thought he was not too bad ‘more mileage in him’… They have stopped a lot of his medications but given him ‘happy pills’ suggesting that a lot of his problems were due to anxiety—he is even walking again…and had made a ‘miraculous recovery.’ (*Carer for P10, field notes Centre A*)
  - It was an admission avoidance to an inappropriate environment…I mean he has several different issues, the main issue for him has recently been regarding his kidneys and his catheter and sorting that side of things out. And then he's had a pace maker in to try and, you know, stabilise his atrial fibrillation but actually to improve his cardiac output…to prevent him from going to bradycardic…The problem with him is that he's got a variety of different problems, and if you give him a diuretic to try and alleviate his fluid retention he goes into crashing hypernatremia…Even with a small dose of furosemide he'll then suddenly become quite confused if you're not careful… So the hospice were great because they took him out of the home scene and…looked after him, stopped much of his medication and put him on an antidepressant…P.10 is, from a medical point of view, is much more, erm, on an even keel…he was really getting…a bit depressed around at the end of last year…I think having some sort of respite for himself really, helped no end. (*HP4, interview GP to P10 Centre A*)

As a condition heart failure has a poor prognosis however, recognising the end-of-life stage can be difficult. Patients may experiences periods of acute exacerbation of symptoms interspersed with periods of relative stability which can make forward planning difficult (box 4C).

The complexity and uncertainty involved in the management of heart failure at end of life was illustrated in an avoided hospital admission in favour of hospice care, for one frail participant. The person-centred care provided by the hospice had some surprising, positive outcomes for this patient (box 4D). The hospice were able to attend to the needs of the whole person, including mental health needs, and provided care better suited to patients who are approaching the end of their lives.

**Figure 2:**
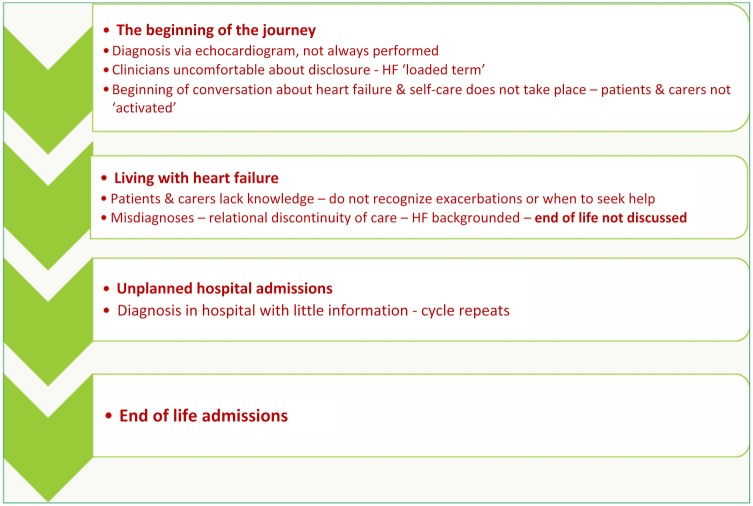
Points in patient pathways where risk of hospital admission is increased.

Difficulty in identifying end of life in patients with heart failure contributed to a lack of discussion and forward planning. Furthermore, the availability of community-based resources around end-of-life care made this a point in the pathway where patients were at particular risk of unplanned admissions and of dying in hospital (figure 2).

## Discussion

This study identified how challenging it is to provide care for people with complex healthcare needs in a complex healthcare system. In all three of the study areas fragmented services and poor managerial continuity created barriers to care and increased the risk of hospital admission for heart failure. Different service providers and professional groups had unintentionally cocreated structures, systems and professional hierarchies that militated against the provision of seamless care. Individual clinicians tried to override organisational anomalies so that patients could access the specialist services they needed. Not all of these attempts were successful.

We identified a number of points in patient pathways where risk of hospital admission for heart failure is increased (figure 2). In some cases, GPs did not make accurate diagnoses, recognise exacerbations, optimise care or were unable to access specialist nurse services for their patients owing to lack of service provision. Some GPs were reluctant to refer patients to specialist nurses either because they wanted to manage the patient themselves or they had little knowledge of the service. In these instances, relational continuity of care with the same GP was not always beneficial.

A quality statement in NICE guidelines for heart failure recommends people with chronic heart failure should be offered “personalised information, education, support and opportunities for discussion throughout their care to help them understand their condition…”^40^ Lack of diagnoses and explanation resulted in patients having little awareness of heart failure or when to seek help. We argue that this factor increased the risk of unplanned admissions. Although heart failure specialist nurses were particularly praised for educating and supporting people with heart failure, patient access to this service was limited. Financial and organisational constraints created variations in the provision of specialist nurse services across the three research centres. In one study centre NHS commissioners utilised evidence from NICE guidance to restrict the provision of community heart failure nurse services to patients with left ventricular systolic dysfunction only (LVSD). Therefore, patients without LVSD may not have received the education and self-care support needed to help avoid (costly) unplanned admissions.

Provision of end-of-life care appeared to be compromised by clinical uncertainty, poor clinician/patient communication, lack of advance care planning and availability of appropriate resources in the community.

Besides giving physical, emotional and practical support, most carers took on an ‘ambassadorial’ role in relation to the people they cared for. Carers chased up lost medical notes/letters and played a key role in unravelling problems caused by poor communication and co-ordination across fragmented health-care systems. As carers gained experience they became more proactive in challenging the organisation and provision of care on behalf of the person they cared for. In this respect carers shouldered a lot of stress and responsibility, even though most of them had their own health problems to deal with.

### Strengths and limitations of this study

A strength of this study was the ethnographic approach. Data was collected in real time, rather than through recall of events. Methodological strength was achieved through triangulation of three contrasting localities; three researchers; complementary qualitative methods, patient and carer experiences and clinician perspectives across different service sectors.

A limitation of this study was the small sample size of 31 patients, which may not represent the diversity of people with severe heart failure. However, we feel the transferability of our findings is demonstrated by agreement with the existing literature. An observer effect resulted in some patients reporting (positive) changes in clinician behaviour during observation. However, patients provided comparative descriptions of preobservation care. A further limitation is that remote monitoring and support models (telemonitoring or telephone support) were not widely utilised in the care settings where the research was undertaken which some health systems are beginning to adopt. The impact of these technologies on unplanned admissions is uncertain.

### Comparison with other studies

Our findings are consistent with published literature. Barriers to diagnosing and managing heart failure in primary care have been identified in earlier qualitative studies.^27^ ^29^ ^41^ ^42^ A recent qualitative study concluded that nothing had changed over the past 10 years in barriers to accurate diagnosis and effective management of heart failure, in addition uncertainty about end-of-life care was reported.^43^ The ‘back-grounding’ of heart failure leads to misdiagnoses^44^ and difficulties around the terminology ‘heart failure’ creates barriers to disclosure.^45^

Both education and self-management programmes for heart failure patients have been shown to reduce heart failure and all cause hospitalisations, with a ‘dose’-related effect^11^ and a number of studies have linked rehospitalisation with failed self-care.^46^ Numerous studies have also identified a lack of patient understanding of heart failure^25^ ^47^ including a lack of patient knowledge of medications and self-care.^44^ ^46^ ^48–51^ A need for more patient/clinician communication and information, especially at diagnosis and nearing end of life was identified in a number of studies.^25^ ^45^ ^52–54^ A qualitative study that examined reasons for readmission in heart failure found these could have been prevented if patients requested help earlier and if multidisciplinary professional help were available.^55^ A previous study on unplanned hospital admissions, by the same authors found services designed around existing systems and professional hierarchies, rather than the needs of patients, were a driver for unplanned admissions.^56^ A new finding from our current study is a possible link between poor managerial and relational continuity of care and unplanned admissions.

The reduction of hospital admissions, for conditions such as heart failure, requires system integration between primary and secondary care. The current barriers encompass lack of patient knowledge, diminishing primary care co-ordination and specialist care that needs to be available outside of acute hospital care in the community. While innovations such as telemonitoring are being developed to support these integration gaps, on their own they are unlikely to overcome system barriers which were illustrated by patients in our study.

The data supporting the efficacy of telemonitoring on reducing hospital admissions is mixed. Recently reported clinical trials of telemonitoring have not demonstrated the positive impact on hospital admissions found from a systematic review based on smaller studies.^57–59^

While in our study, patient participation was directly related to heart failure admissions; the analysis also showed that comorbidity was an important factor in these hospital admissions, and which is supported by current evidence. Comorbidity creates a clinical barrier which hinders acute heart failure management and further developments need to consider how this critical issue is addressed.^60^

### Implications for clinicians and policymakers

Healthcare delivered across multiple services, confusion about eligibility for specialist heart failure services and relational/managerial discontinuity of care added additional complexity and likelihood of suboptimal management and unplanned admissions. A lack of timely and accurate diagnosis of exacerbations resulted in unplanned admissions. Although heart failure is a complex and challenging condition to detect and manage in primary care, the need for further learning about this condition and specialist services, was recognised. Explanation and education are often missing first steps for the patient, reducing the likelihood of acceptance and learning to self-care/monitor. Patient education should be seen as an ongoing ‘conversation’ with trusted clinicians and end-of-life planning should be broached within this context.

### Unanswered questions and future research

More work is necessary to improve access to appropriate and timely care for people with chronic heart failure. Services should be commissioned that give a unified, integrated and simple to access system, regardless of the patient's home locality. The impact of these services should be evaluated for patient outcomes including unplanned admissions. All patients should have access to multidisciplinary heart failure teams and specialist nurse services when needed. All patients should receive ongoing monitoring and management of heart failure in line with evidence-based clinical guidelines.^7^ ^61^

The impact of increasing the confidence, knowledge and skills of general practitioners in managing patients with heart failure should be considered.
